# Supplementary material for: DeeReCT-APA: Prediction of Alternative Polyadenylation Site Usage Through Deep Learning
Source: Genomics Proteomics Bioinformatics. 2021 Mar 2;20(3):483–95. doi: 10.1016/j.gpb.2020.05.004 (PMC9801043; doi:10.1016/j.gpb.2020.05.004)
Supplement: Supplementary File S1 — Supplementary materials for DeeReCT-APA [file mmc1.pdf]

File S1 Supplementary Materials for DeeReCT-APA: Prediction of Alternative Polyadenylation Site Usage Through Deep Learning

Zhongxiao Li[1], Yisheng Li[2], Bin Zhang[3], Yu Li[1], Yongkang Long[1,2], Juexiao Zhou[2], Xudong Zou[2], Min Zhang[2], Yuhui Hu[2,*], Wei Chen[2,*], and Xin Gao[1,*]

[1]*King Abdullah University of Science and Technology* (KAUST)*, Computational Bioscience Research Center* (CBRC)*, Computer, Electrical and Mathematical Sciences and Engineering* (CEMSE) *Division, Thuwal, 23955-6900, Saudi Arabia.*

[2]*Department of Biology, Southern University of Science and Technology* (SUSTech)*, Shenzhen, 518055, China.*

[3]*Cancer Science Institute of Singapore, Singapore 117599, Singapore*

[*]All correspondence should be addressed to Yuhui Hu (huyh@sustech.edu.cn) Wei Chen (chenw@sustech.edu.cn) and Xin Gao (xin.gao@kaust.edu.sa)

S1 Feature extraction in Feature-Net

We in this section give a list of all the features extracted from raw sequence by the Feature-Net. A list of feature types and their dimensions are shown in Table S1. We also list out the polyadenylation signals, the AUE, CUE and ADE elements, as well as RBP motifs that are used:

Polyadenylation Signals: AATAAA, ATTAAA, TATAAA, AGTAAA, AAGAAA, AATATA, AATACA, CATAAA, GATAAA, AATGAA, TTTAAA, ACTAAA, AATAGA, AAAAAG, AAAATA, GGGGCT, AAAAAA, ATAAAA, AAATAA, ATAAAT, TTTTTT, ATAAAG, TAAAAA, CAATAA, TAATAA, ATAAAC

AUE Elements: GGGGAG, GUGGGG, GGGUGG, UUUGUA, GUAUUU, CUGUGU, UAUAUA, AUAUAU, UUUAUA, UGUAUA, AUGUAU, UGUAUU

CUE Elements: UAUUUU, UGUUUU, UUUUUU, AAUAAA, AUAAAG, AAAUAA

CDE Elements: GUGUCU, CUGCCU, UGUCUC, UUAUUU, UUUCUU, UGUUUU, UGUGUG, GUGUGU, CUGUGU, CUGGGG, UGUCUG, GUCUGU

ADE Elements: CCUCCC, CUCCCC, CACCCC, CCCGCC, CCCCGC, CCCGCG, GGUGGG, GGCUGG, GGGUGG, GGGCAG, GGCCAG, GGGGCC, GGGAGG, GGAGGG, GGGGAG

RBP Motifs: UUUUAU, GGGAGG, GGAGGG, GCUUGC, YGCY, YGCUKY, ARAAGA, UUUUCU, UCAY, CCWWHCC, CCYYCCH, UGGGRAD, GGGA, UKKGGK, GGSKG, UGUA, UGUGU, GAAGAA

S2 The hyperparameters for DeeReCT-APA

We provide a full list of hyperparameters in Table S2. To limit the search space of hyperparameters, we choose to randomly sample hyperparameters that the model is most sensitive to, *i.e.* batch size, learning rate, L2 weight decay and dropout rate and choose the combination that makes the model perform best on the validation set. Note that since we use three different designs for Base-Net, their best hyperparameters are slightly different. Note the best values for hyperparameters that affect regularization of model, *i.e.* L2 weight decay and dropout rate, are higher for more complex model (Multi-Conv-Net), which is reasonable, as complex models are more prone to overfitting and thus their capacity should be limited more by regularization.

S3 Performance of SP parental model and F1 model fine-tuned from SP parental model

As in the main text, we provide the overall performance measures for SP parental models and F1 models fine-tuned from SP parental models for the three different models: DeeReCT-APA, Polyadenylation Code and DeepPASTA (Table S4). The DeeReCT-APA with Multi-Conv-Net shows generally better performance than Polyadenylation Code and DeepPASTA.

We also globally show the quality of prediction of DeeReCT-APA and Polyadenylation Code in F1 hybrid cell using the scatter plot of predicted allelic usage difference versus ground truth allelic usage difference, as in the main text. As it is shown in Figure S2, DeeReCT-APA shows higher correlation between predicted and ground truth allelic usage difference than Polyadenylation Code.

S4 Additional performance measures

In this section, we provide results of some additional tests on DeeReCT-APA's performance. The first is to test the significance of DeeReCT-APA's improvement over existing methods. The second is to use a benchmark dataset other than the one from [1] to evaluate DeeReCT-APA's comparison accuracy.

To test the significance of DeeReCT-APA's improvment over Polyadenylation Code and DeepPASTA, we conducted 5 replicated experiments, where in each of them we independently split the dataset into 5 folds and perform 5-fold cross validation on them. With completely identical settings, we train the model on parental dataset and fine-tune on F1. We report the comparison accuracy of each method in different replicates seperately (Table S5). The t-tests between DeeReCT-APA and Polyadenylation Code as well as DeeReCT-APA and DeepPASTA show that the improvement of DeeReCT-APA over Polyadenylation Code and DeepPASTA is significant (last column).

We also show the performance of DeeReCT-APA on the dataset obtained from [2]. Since this is a multi-tissue dataset, we report the comparison accuracy in each tissue separately. One can see that DeeReCT-APA outperforms existing methods in most of them.

References

[1] Mei-Sheng Xiao, Bin Zhang, Yi-Sheng Li, Qingsong Gao, Wei Sun, and Wei Chen. Global analysis of regulatory divergence in the evolution of mouse alternative polyadenylation. *Molecular Systems Biology*, 12(12):890, 2016.

[2] M. K. K. Leung, A. Delong, and B. J. Frey. Inference of the human polyadenylation code. *Bioinformatics*, 34(17):2889–2898, 2018.
