# Supplementary material for: DeeReCT-APA: Prediction of Alternative Polyadenylation Site Usage Through Deep Learning
Source: Genomics Proteomics Bioinformatics. 2021 Mar 2;20(3):483–95. doi: 10.1016/j.gpb.2020.05.004 (PMC9801043; doi:10.1016/j.gpb.2020.05.004)
Supplement: Supplementary Figure S1 — The structures of DeeReCT-APA models used in the ablation study A. The structure of DeeReCT-APA with interaction layers but without BiLSTM. B. The structure of DeeReCT-APA with interaction layers removed. Comparing A with Figure 1 in the main text, it has BiLSTM removed and only has the affine layer in the interaction layers. In B, the interaction layers are removed altogether and DeeReCT-APA resorted to comparison-based training (to predict which one of the two PAS is of higher usage). Note that an additional affine layer is added on top of the Base Networks to cast the output of the base network (which is a vector) into a scalar. [file mmc2.pdf]

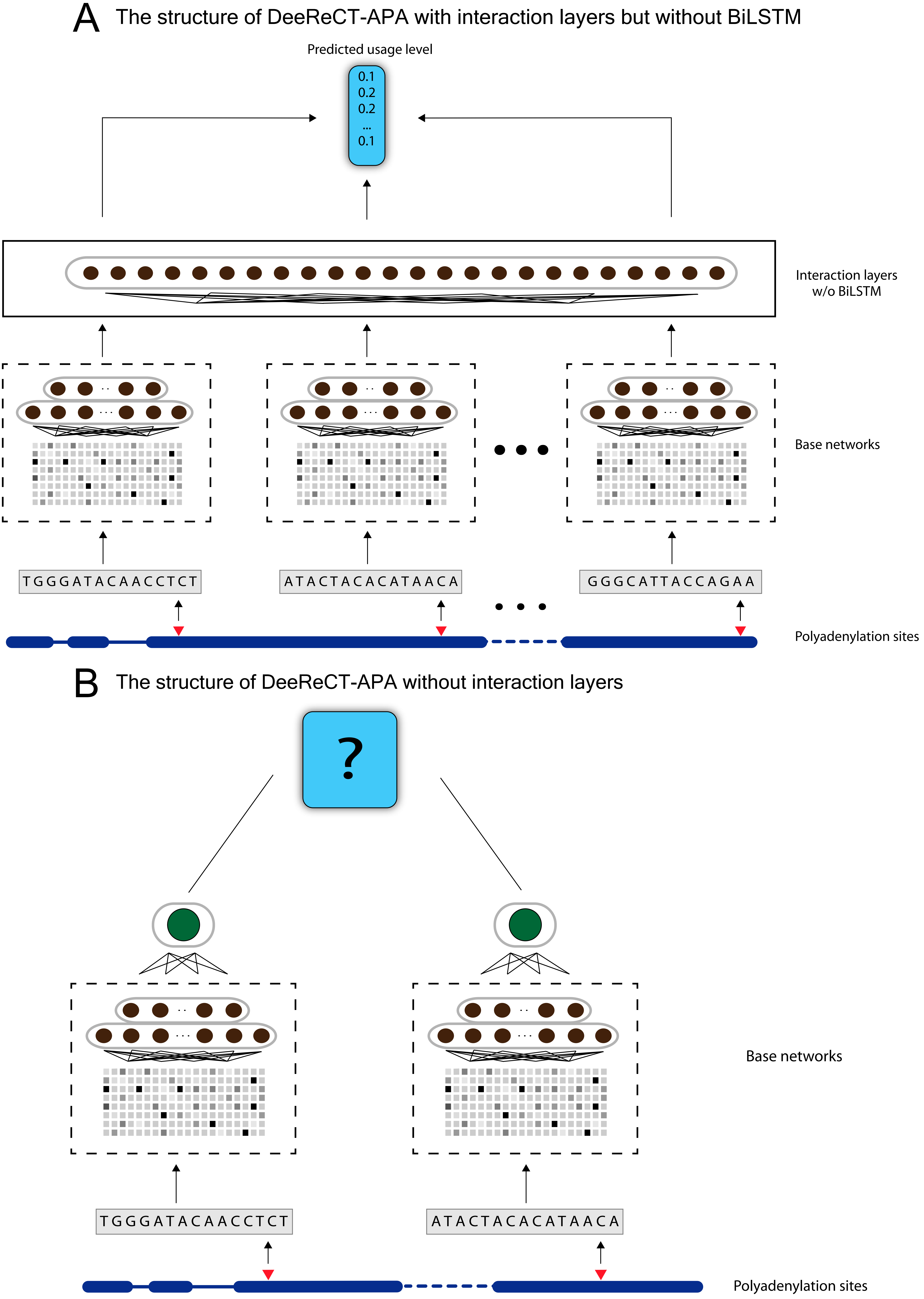

A The structure of DeeReCT-APA with interaction layers but without BiLSTM
Predicted usage level
0.1
0.2
0.2
...
0.1
Interaction layers w/o BiLSTM
Base networks
TGGGATACAACCTCT
ATACTACACATAACA
GGGCATTACCAGAA
Polyadenylation sites
B The structure of DeeReCT-APA without interaction layers
?
Base networks
TGGGATACAACCTCT
ATACTACACATAACA
Polyadenylation sites
