# Supplementary material for: DeeReCT-APA: Prediction of Alternative Polyadenylation Site Usage Through Deep Learning
Source: Genomics Proteomics Bioinformatics. 2021 Mar 2;20(3):483–95. doi: 10.1016/j.gpb.2020.05.004 (PMC9801043; doi:10.1016/j.gpb.2020.05.004)
Supplement: Supplementary Figure S2 — Comparison of the allelic usage difference prediction of DeeReCT-APA and Polyadenylation Code F1 model fine-tuned from SP parental model is used. A. B. The horizontal axis is the ground truth allelic usage value difference between two homologous PAS (which is the BL usage value minus the SP usage value). The vertical axis shows the predicted allelic usage value difference. The scatter plot of DeeReCT-APA is shown in Panel A and Polyadenylation Code is shown in Panel B. As DeeReCT-APA predicts the usage value in percentage, we draw a red line that shows the perfect prediction. C. Pearson correlations between two quantities at different minimum allelic usage difference are shown in the table below. [file mmc3.pdf]

A

DeeReCT-APA's prediction of allelic usage difference

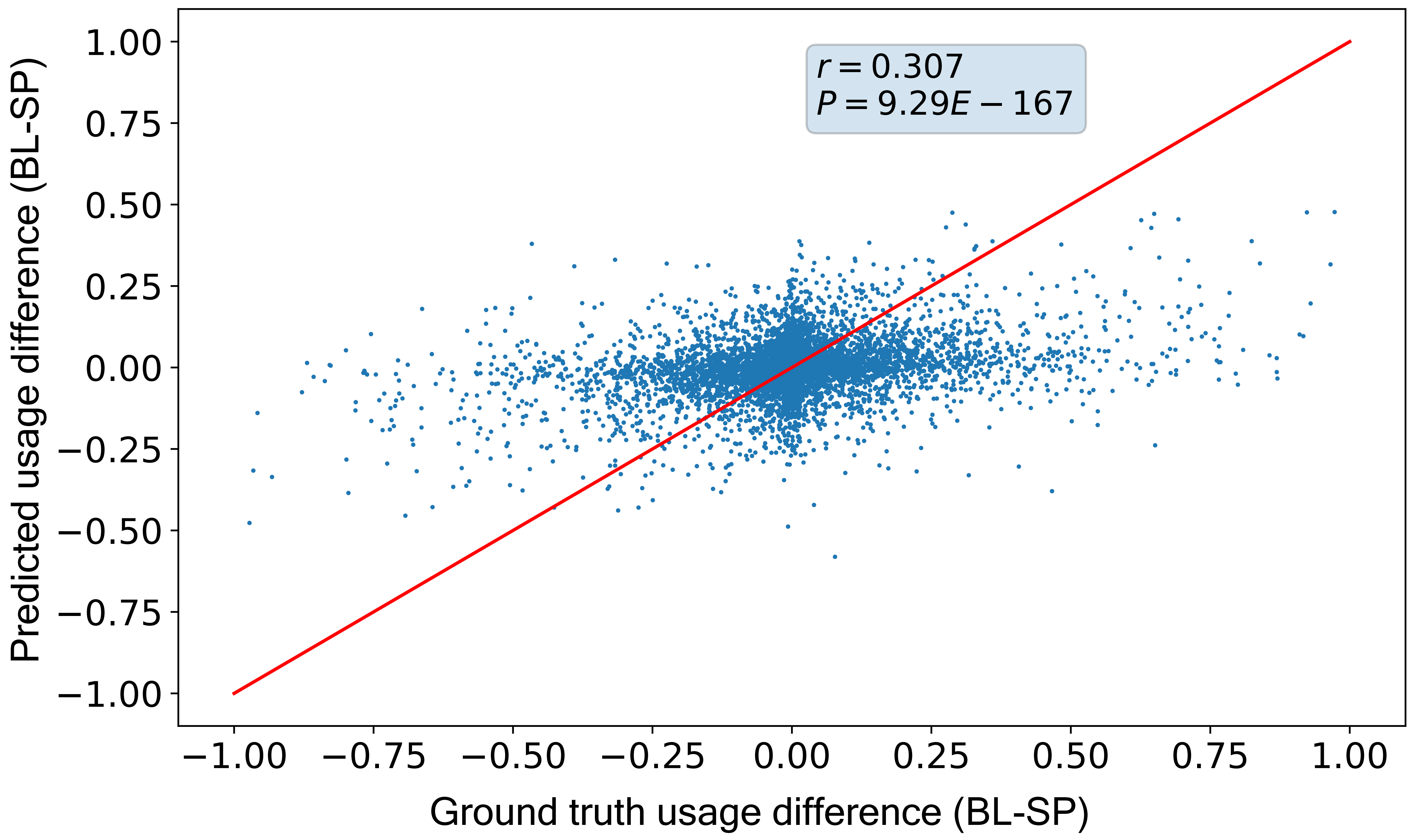

B

Polyadenylation Code's prediction of allelic usage difference

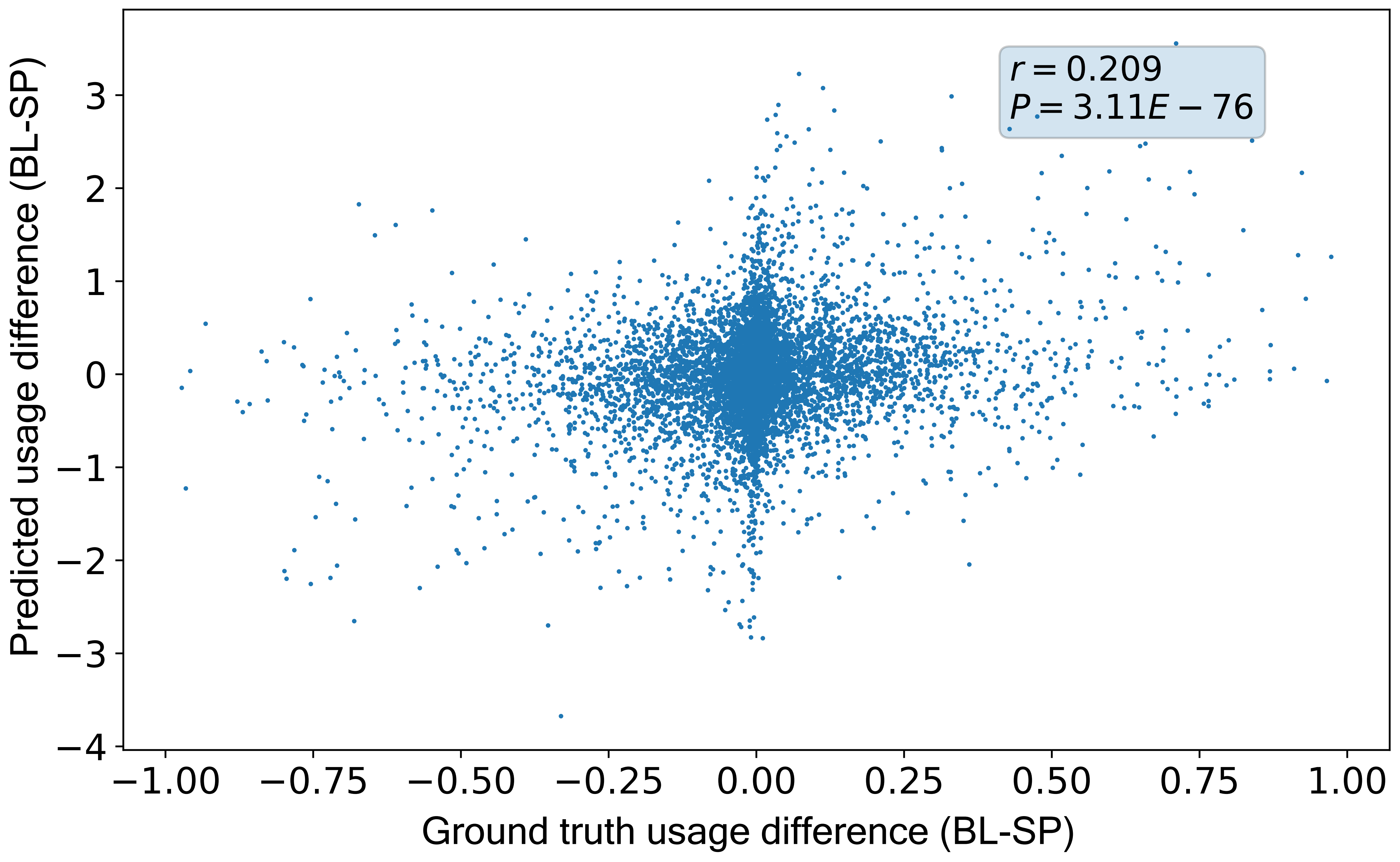

C

PCC (and their *P* values) at different minimum allelic usage difference

Min. allelic usage difference		0.1	0.2	0.3	0.4	0.6	0.8
DeeReCT-APA	PCC	0.400	0.467	0.507	0.536	0.645	0.708
	P value	3.5×10^{-103}	3.5×10^{-74}	4.9×10^{-48}	5.2×10^{-31}	1.5×10^{-16}	4.7×10^{-5}
Polyadenylation Code	PCC	0.307	0.351	0.366	0.402	0.465	0.568
	P value	4.4×10^{-57}	5.3×10^{-39}	2.3×10^{-27}	5.0×10^{-19}	3.2×10^{-9}	7.2×10^{-3}
