# Supplementary figures and images for: DeeReCT-APA: Prediction of Alternative Polyadenylation Site Usage Through Deep Learning
Source: Genomics Proteomics Bioinformatics. 2021 Mar 2;20(3):483–95. doi: 10.1016/j.gpb.2020.05.004 (PMC9801043; doi:10.1016/j.gpb.2020.05.004)

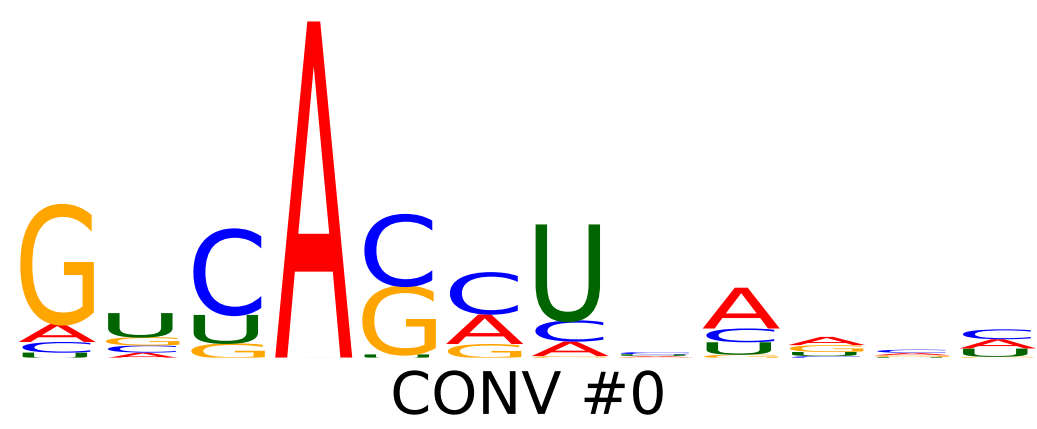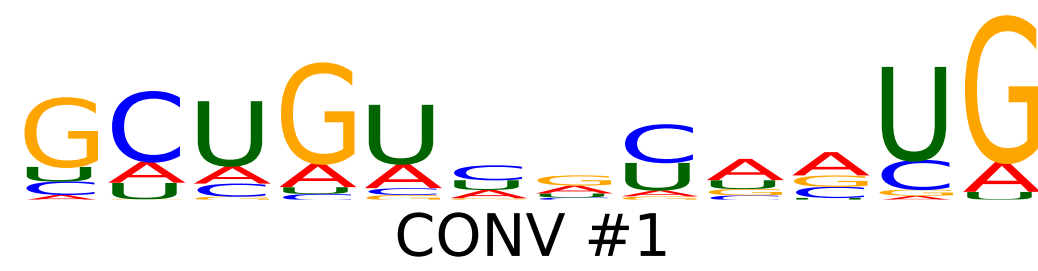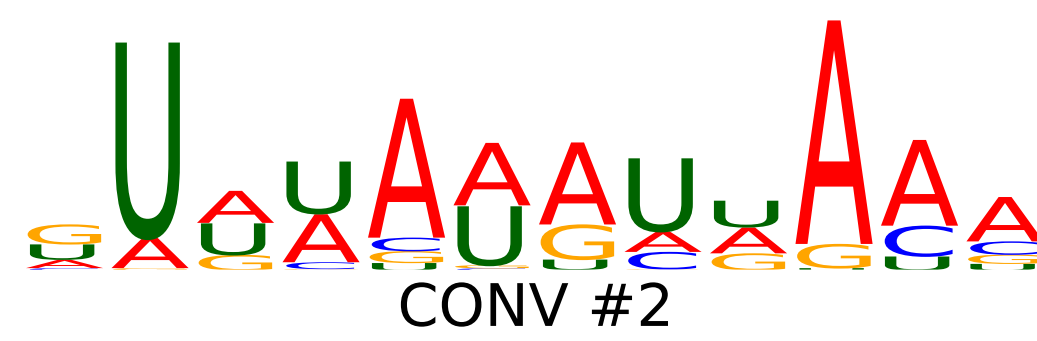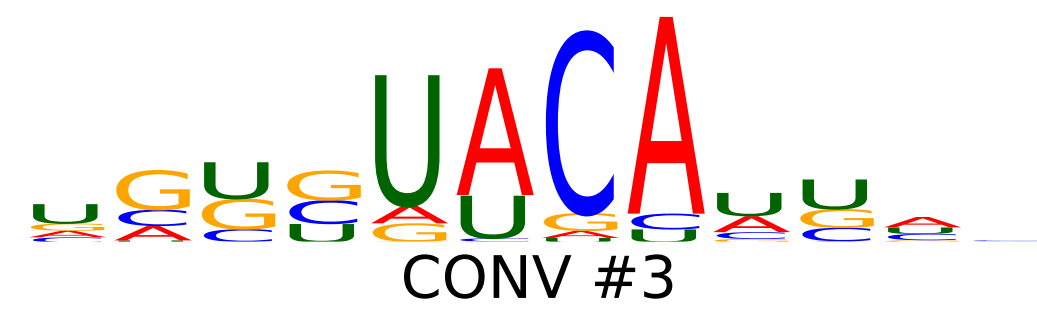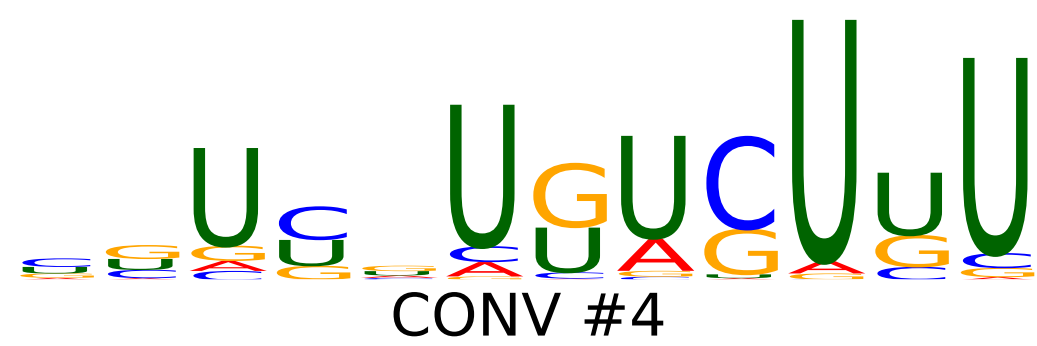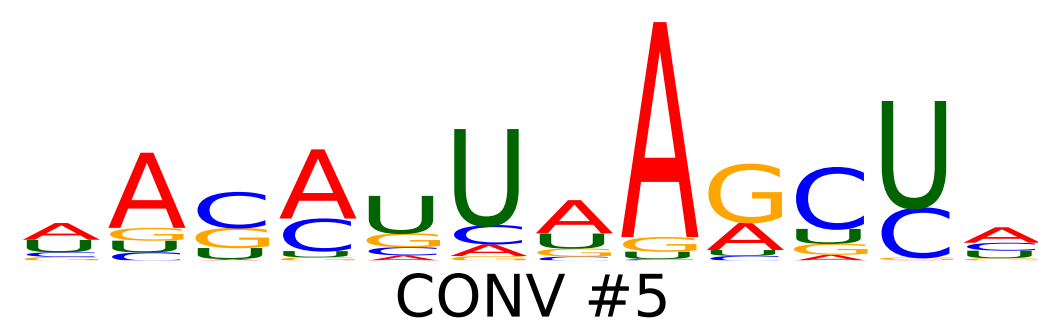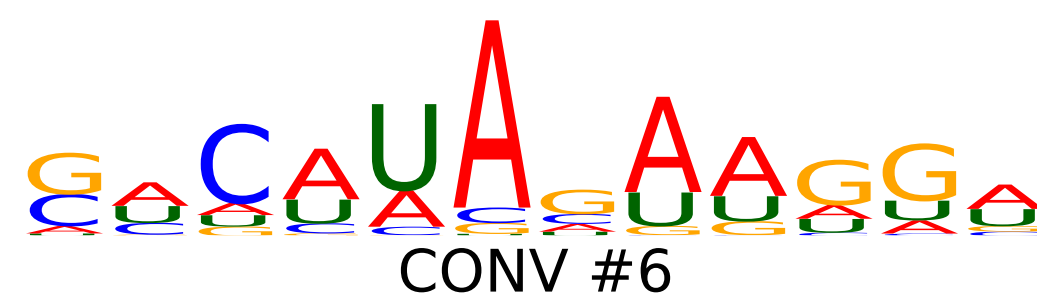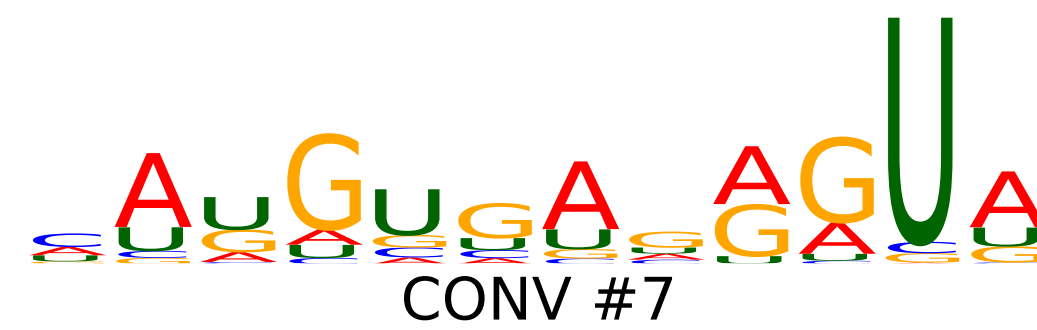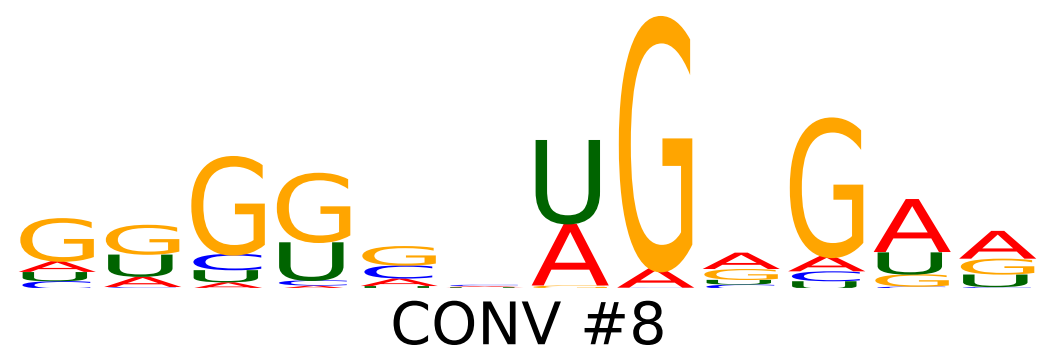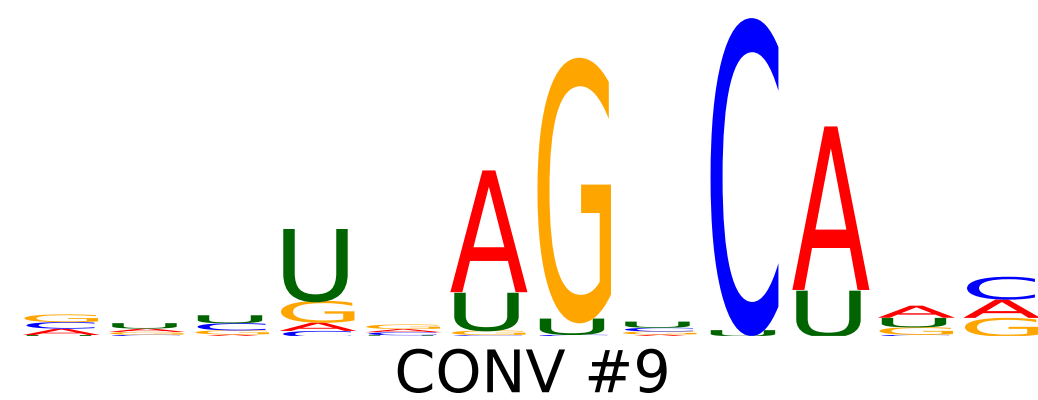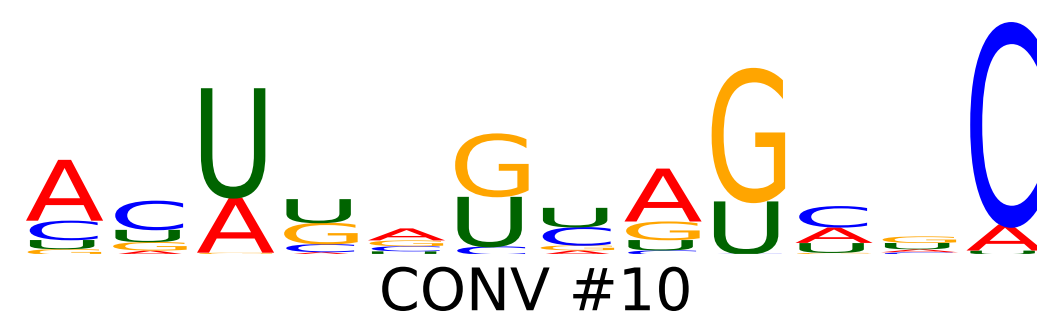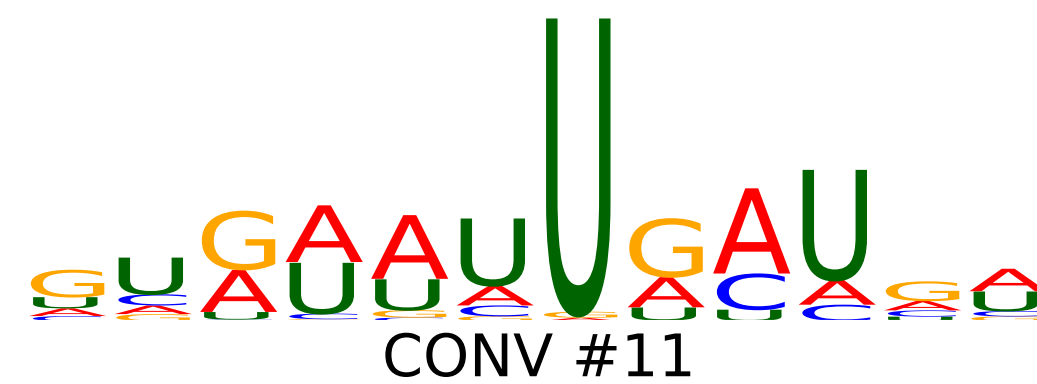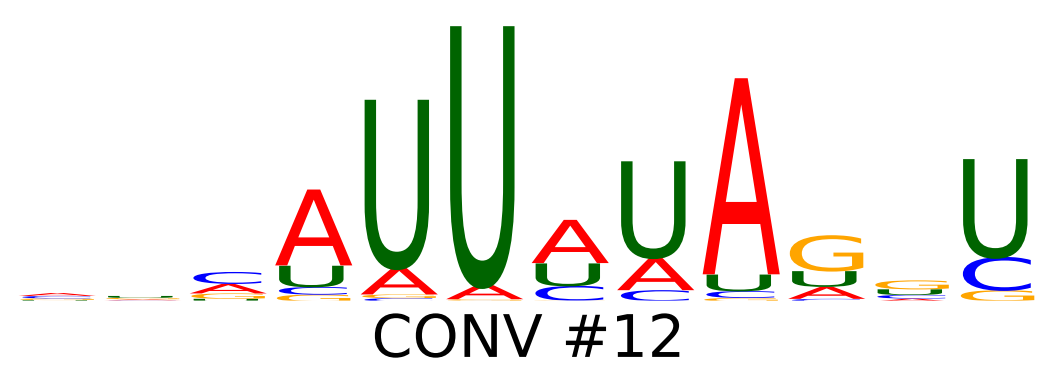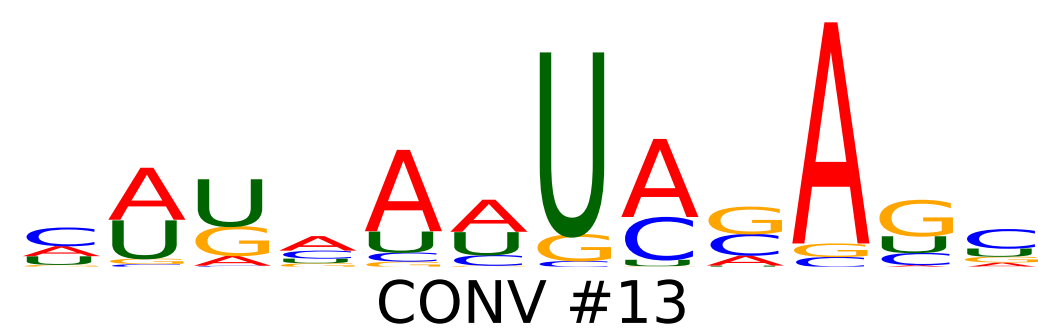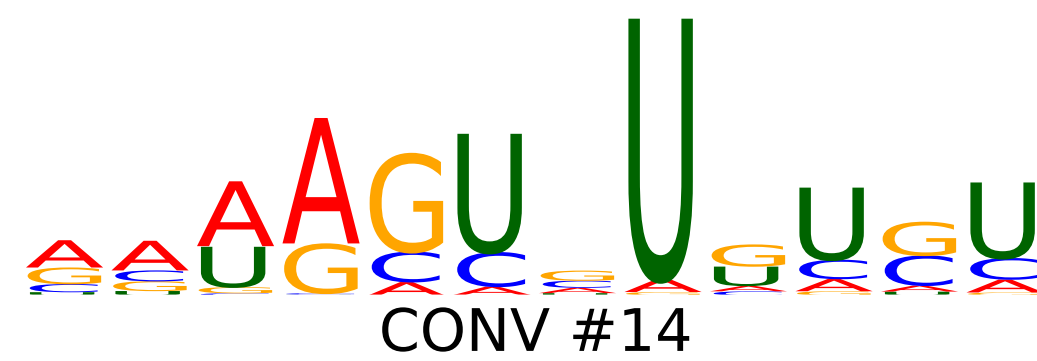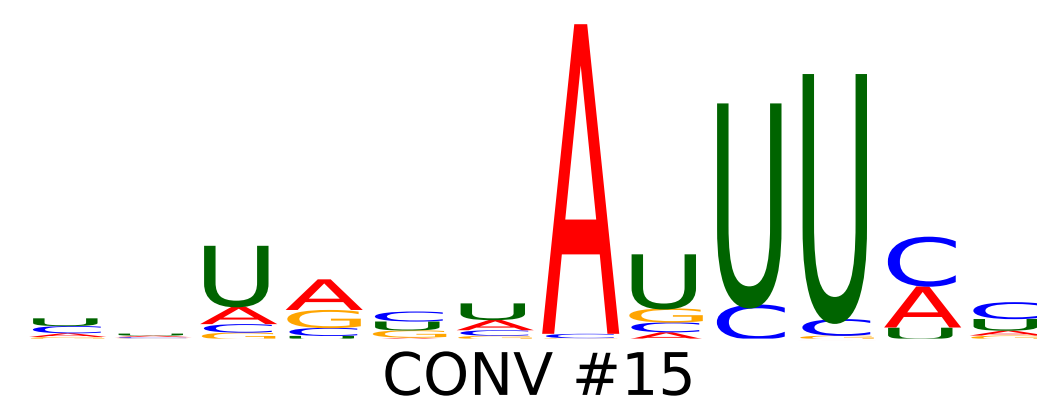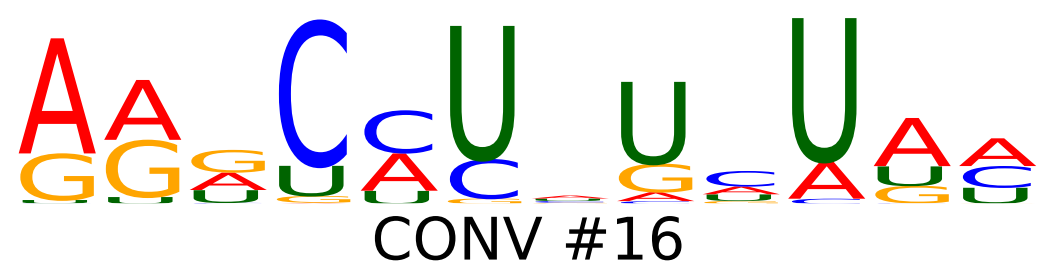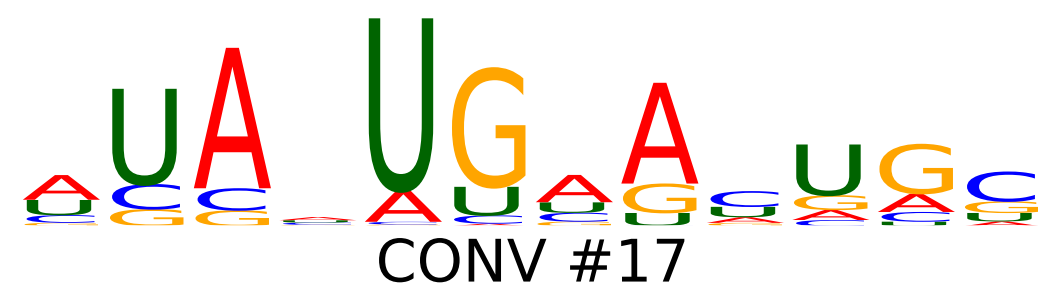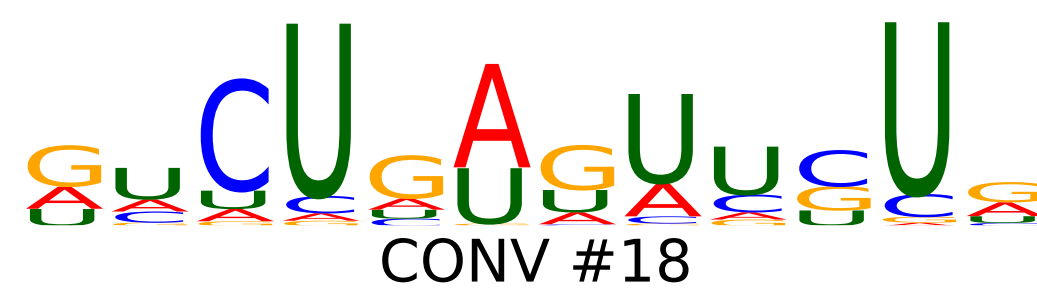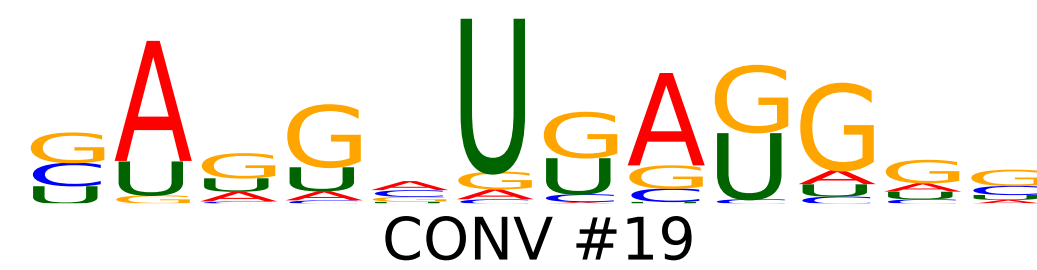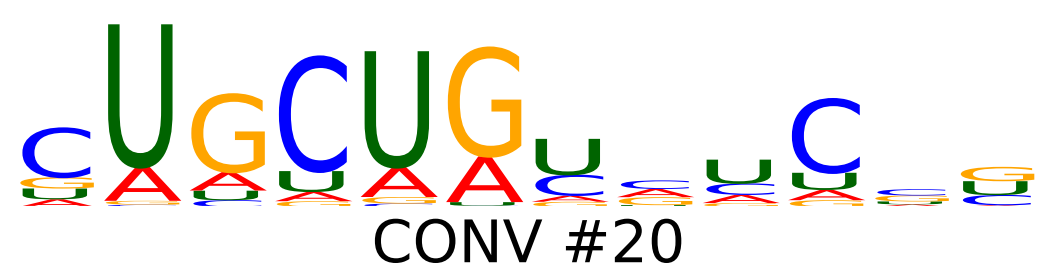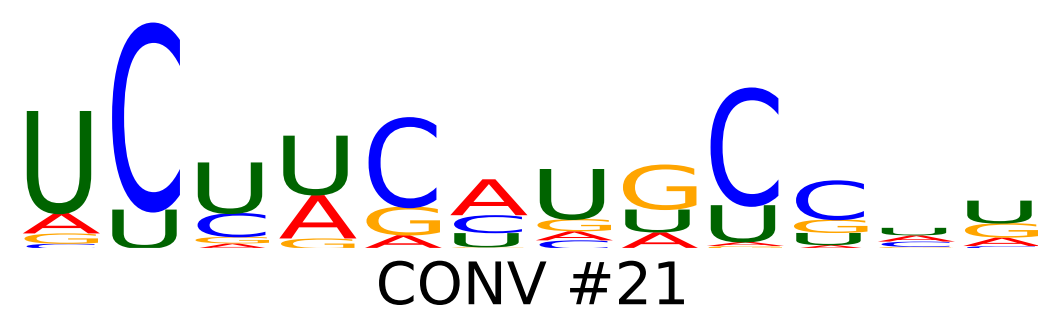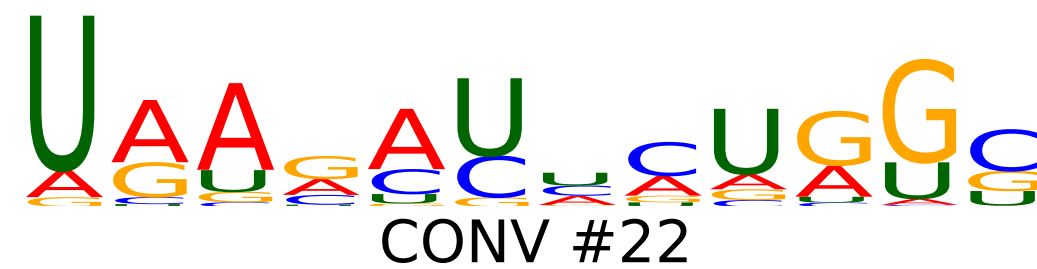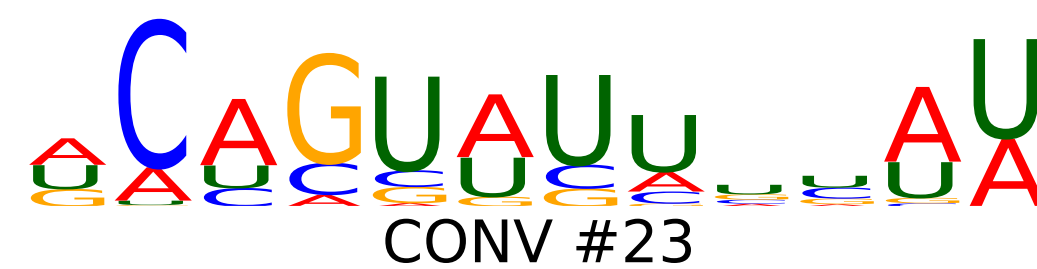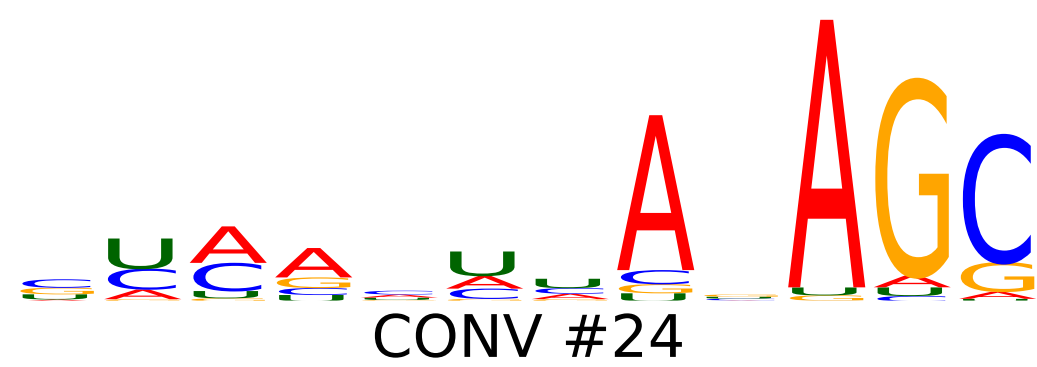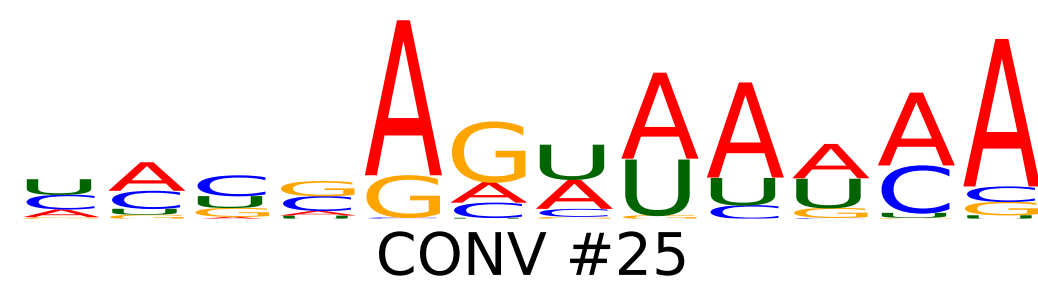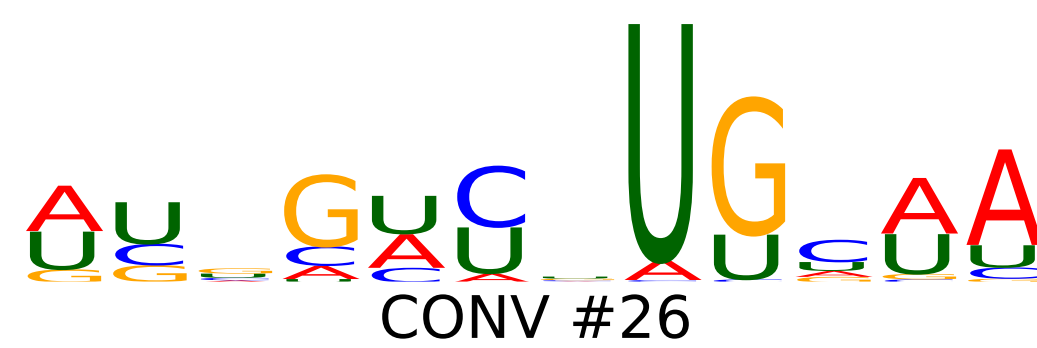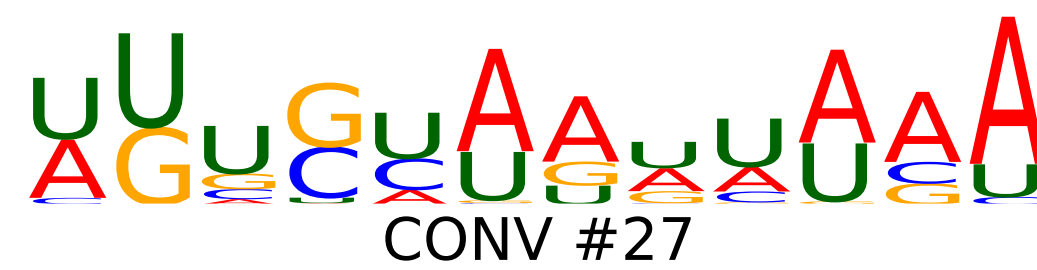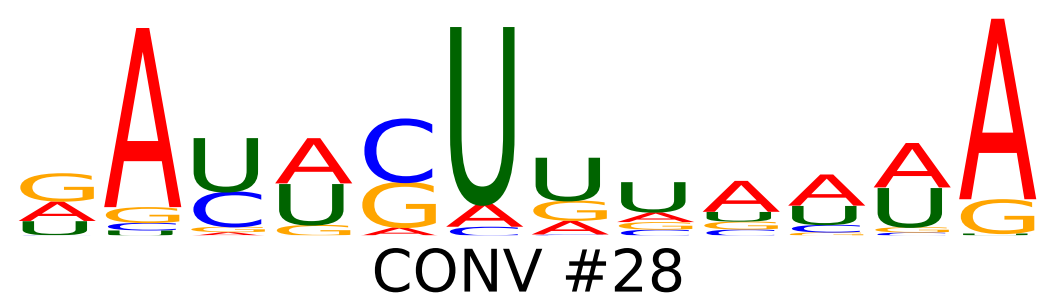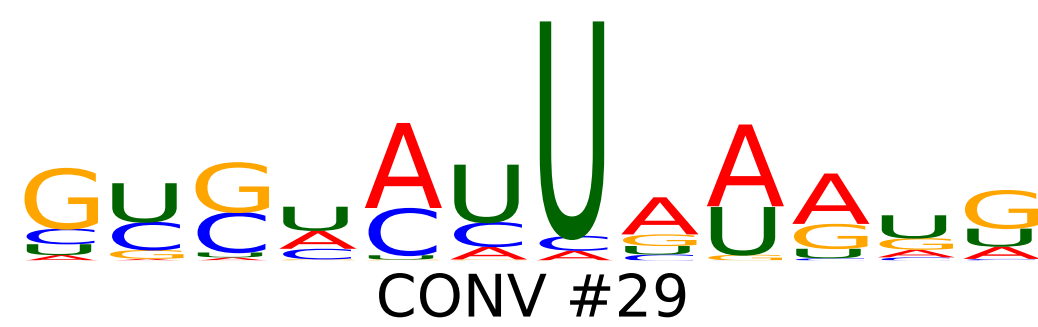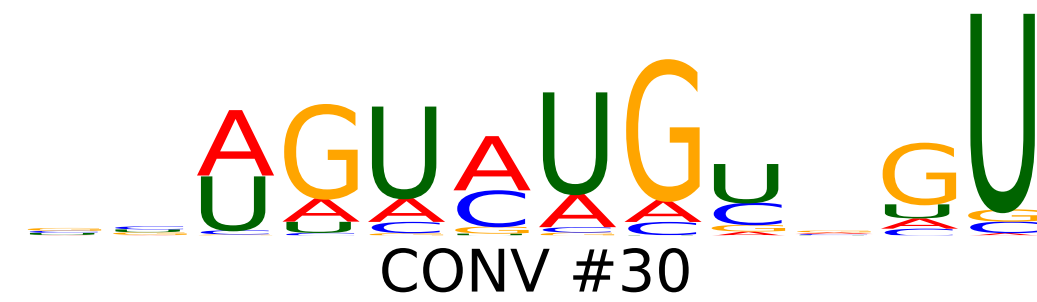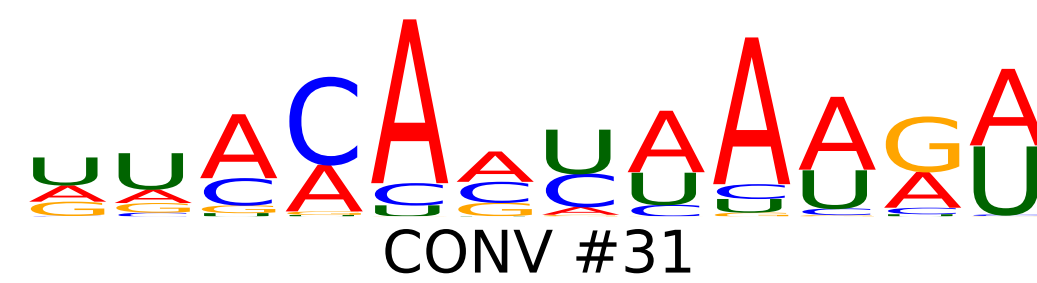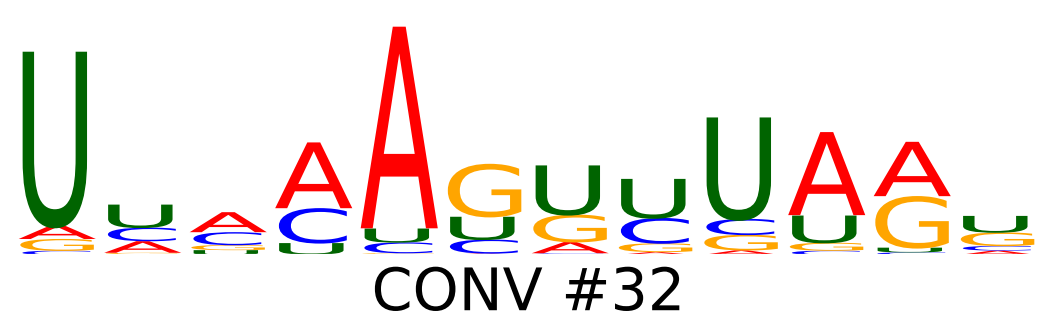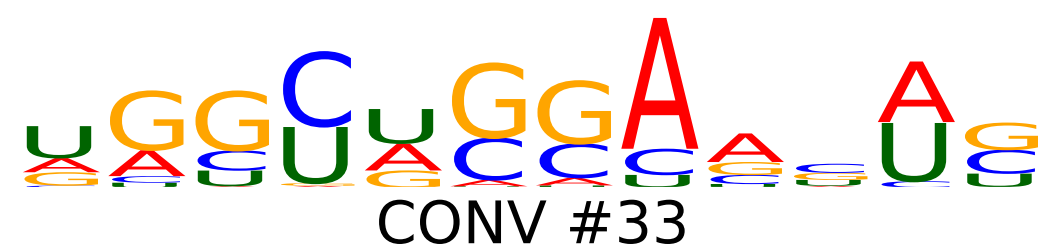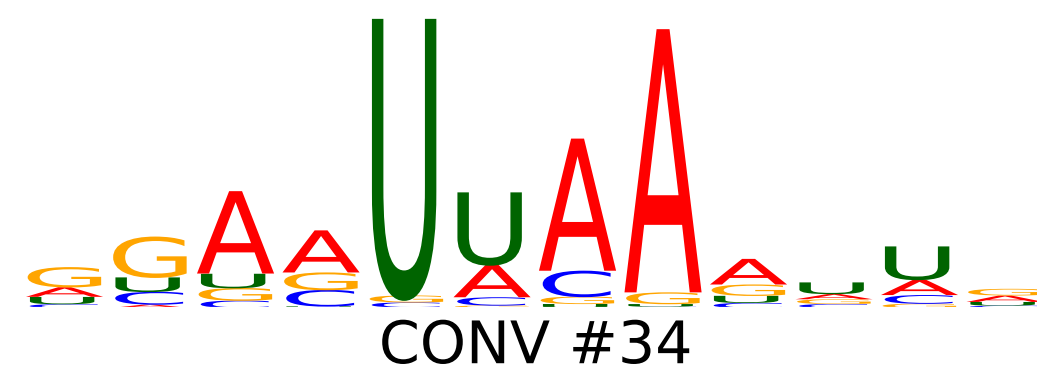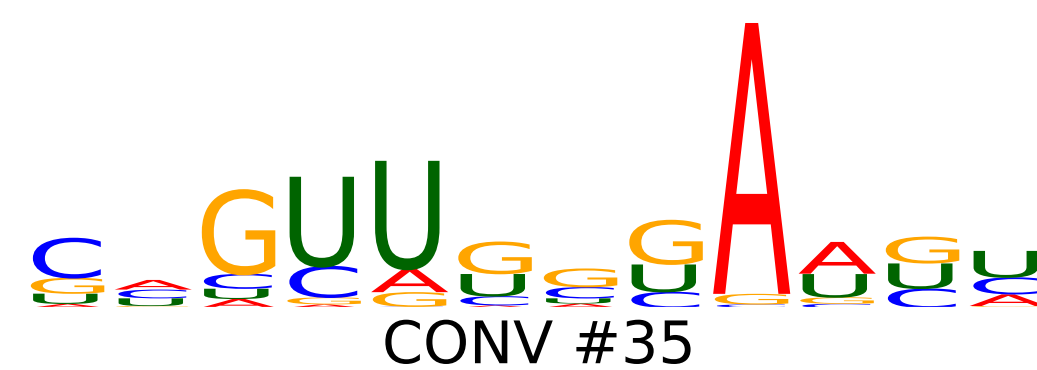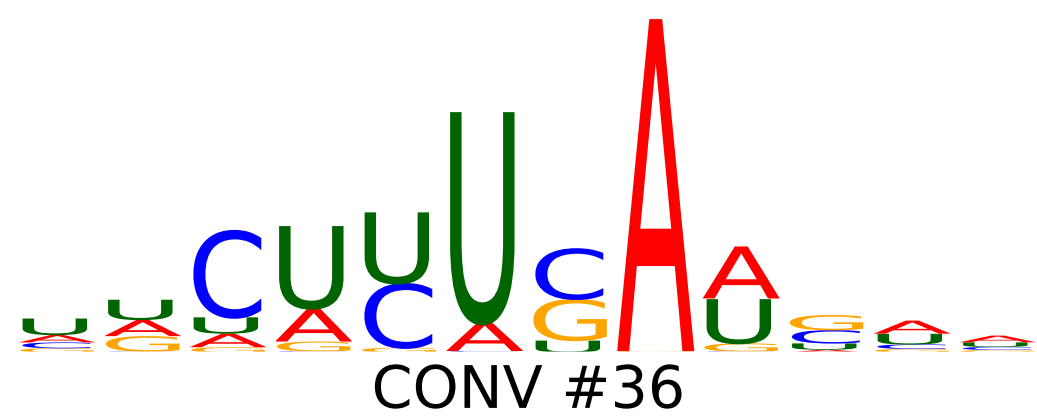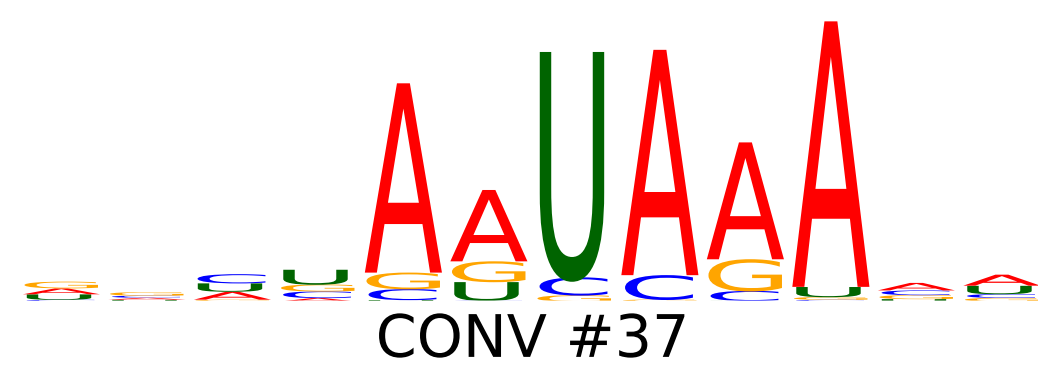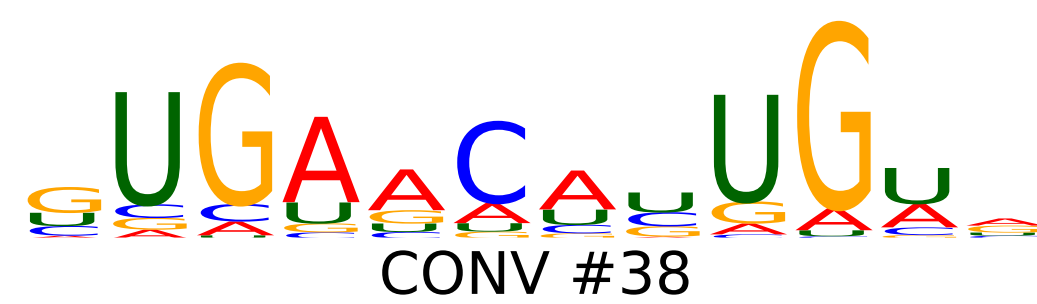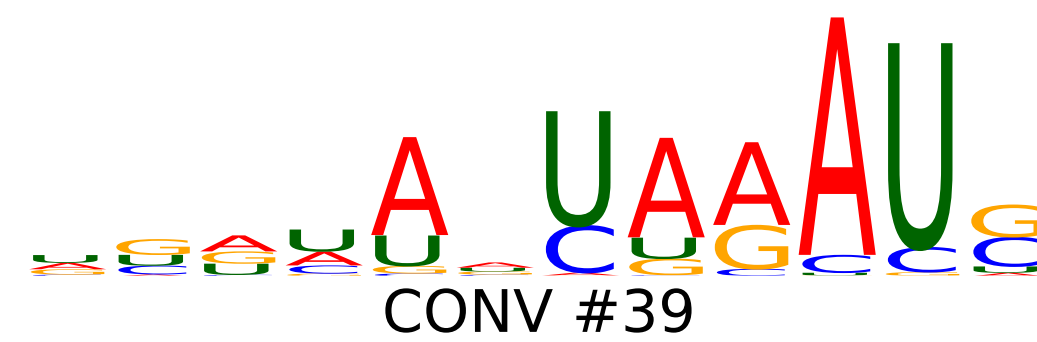

Supplement: Supplementary Figure S3 — Visualization of convolutional filters in layer 1 of DeeReCT-APA There are 40 convolutional filters in layer 1 of DeeReCT-APA. The model is trained on parental BL dataset and fine-tuned on F1. [file mmc4.pdf]

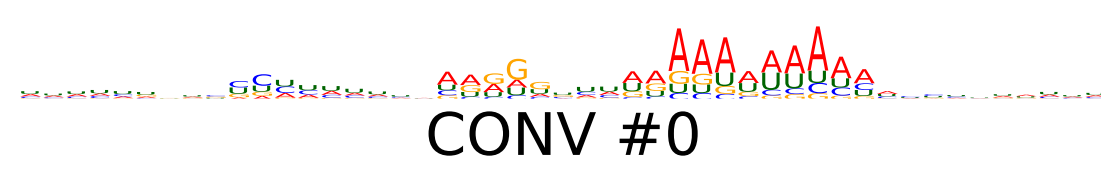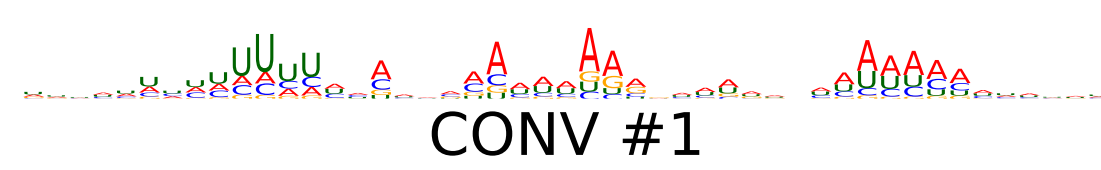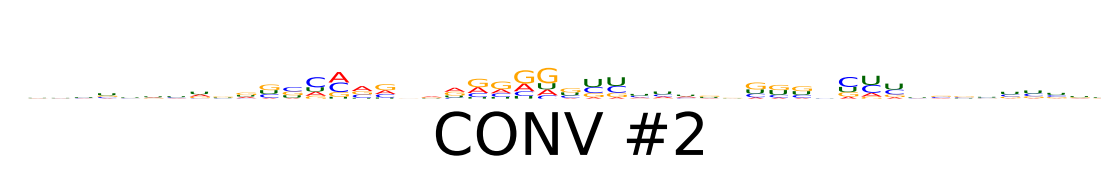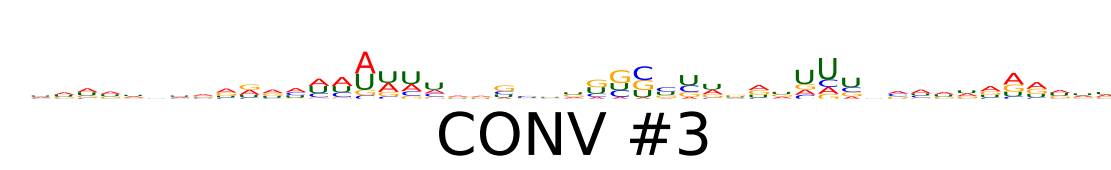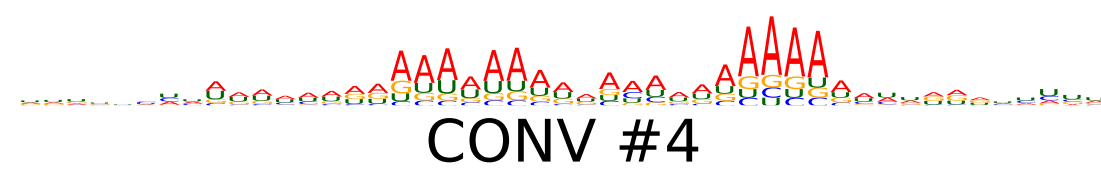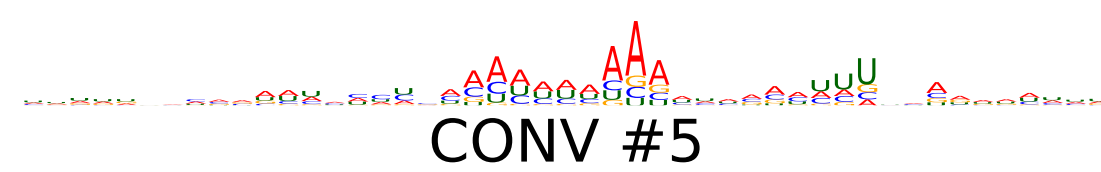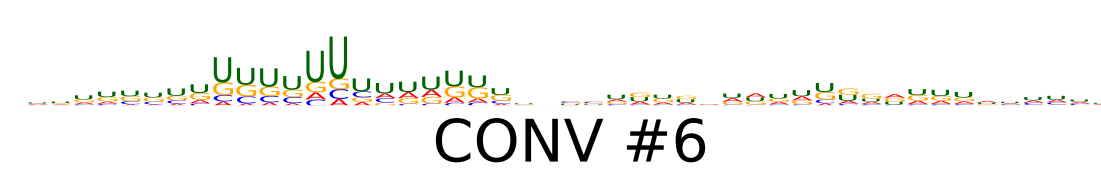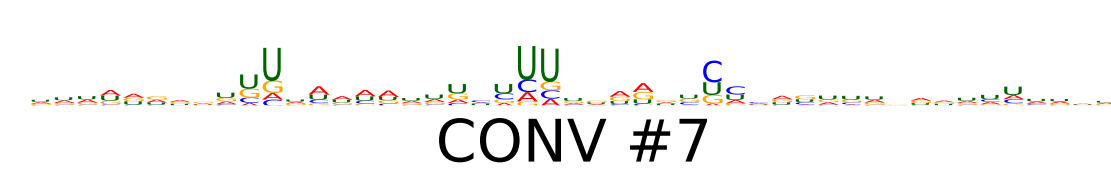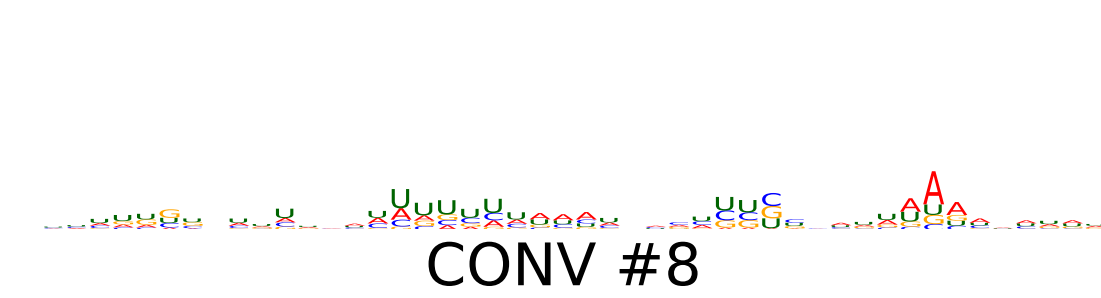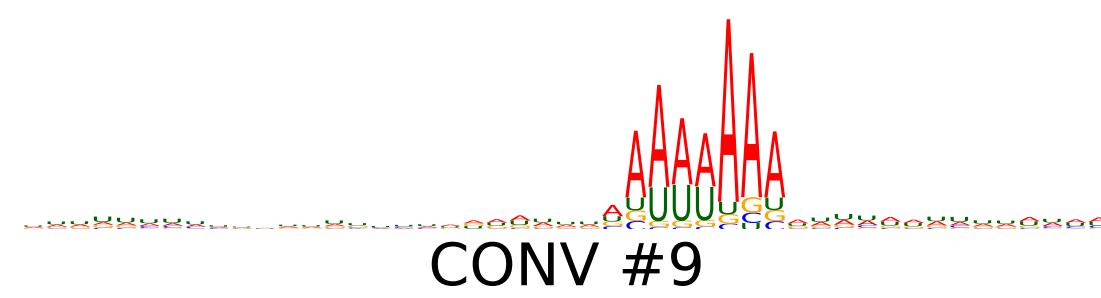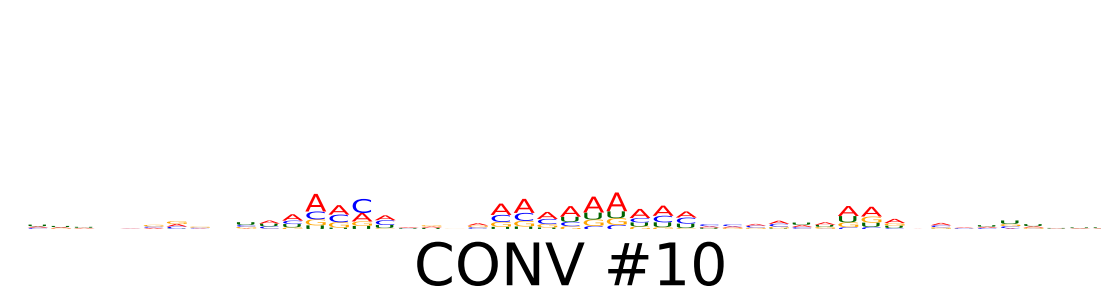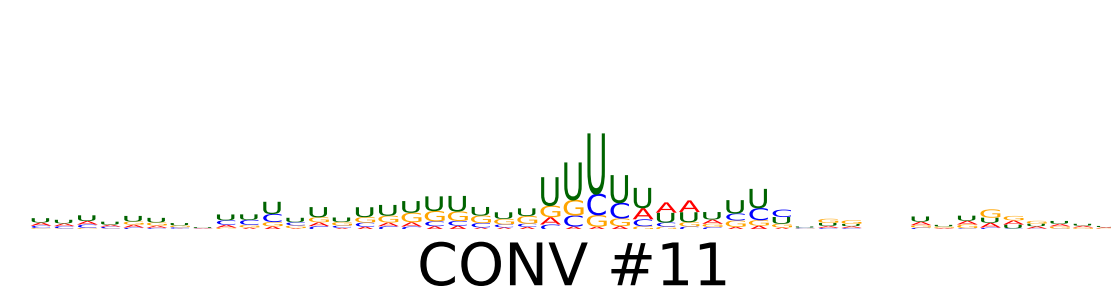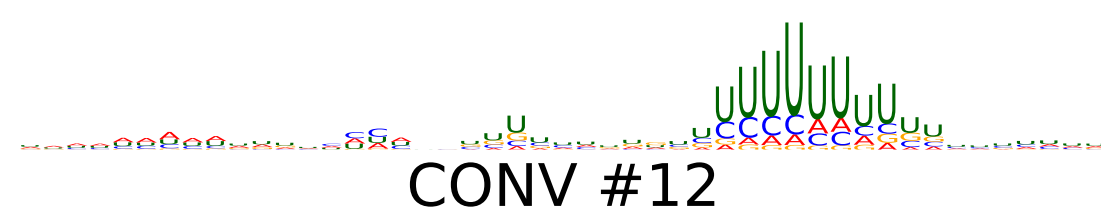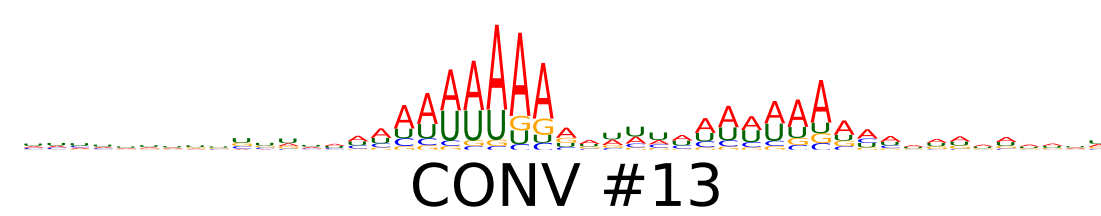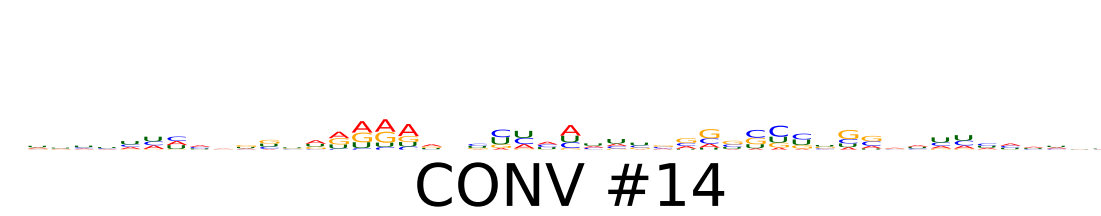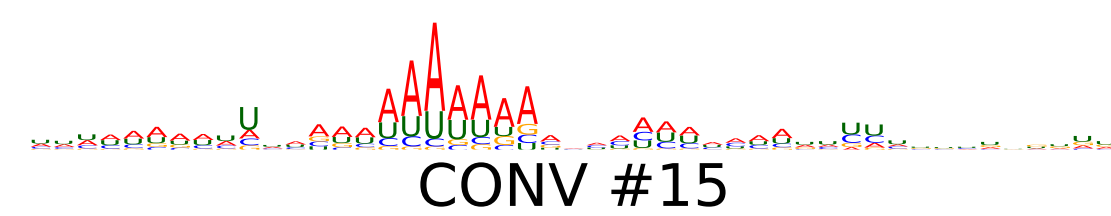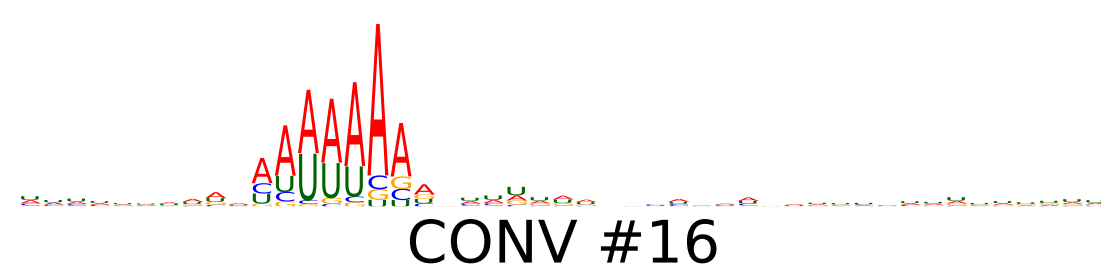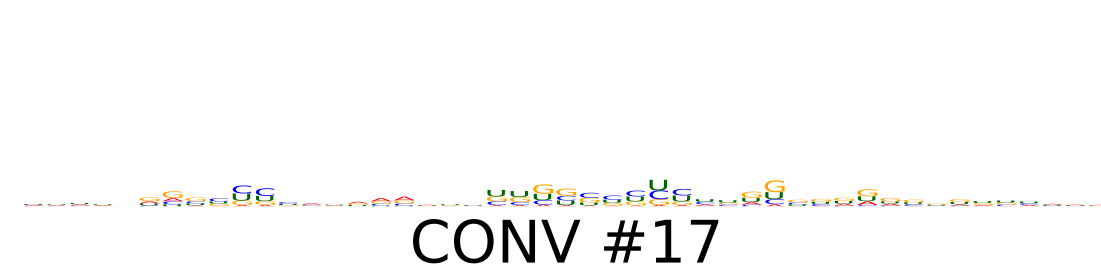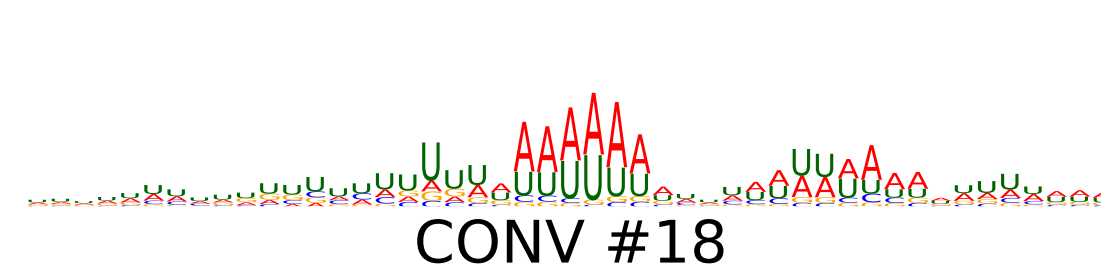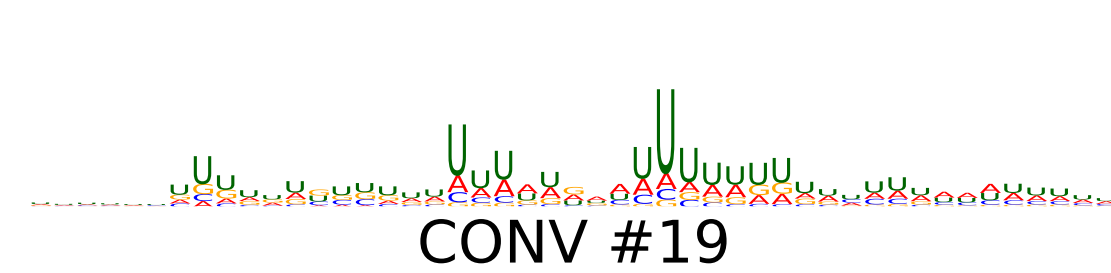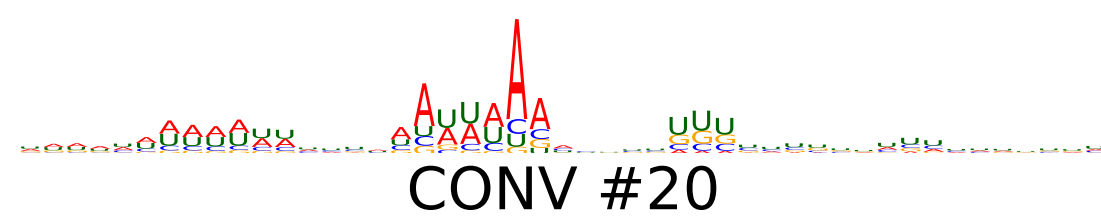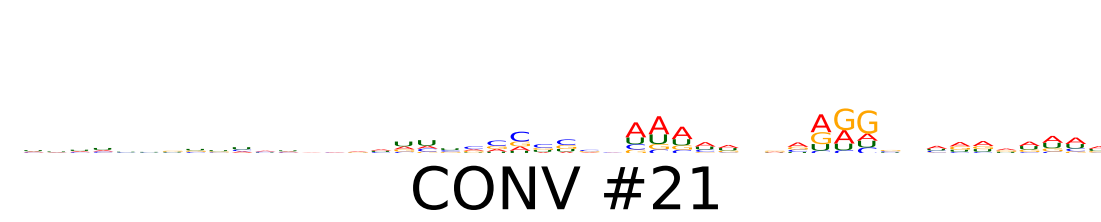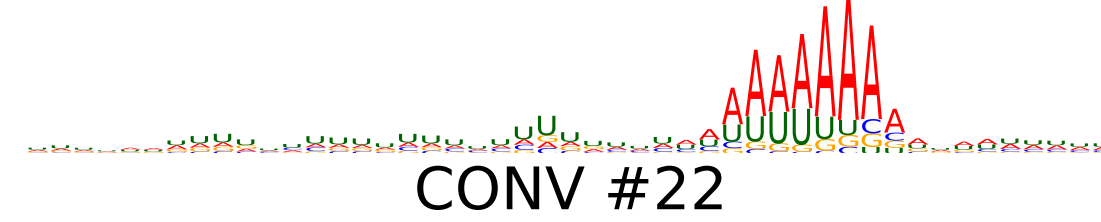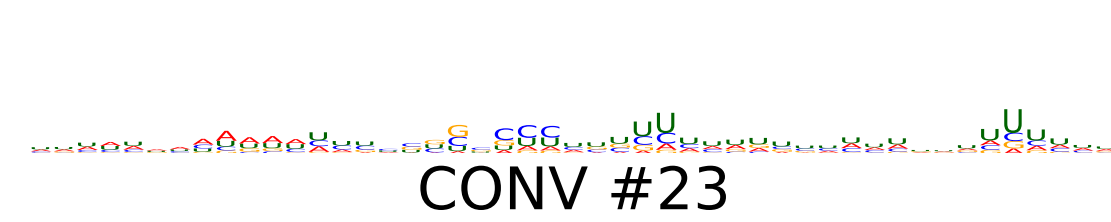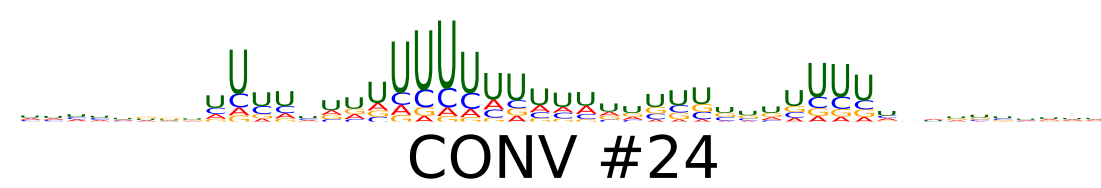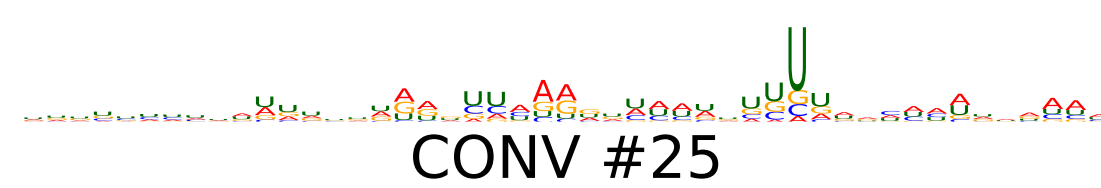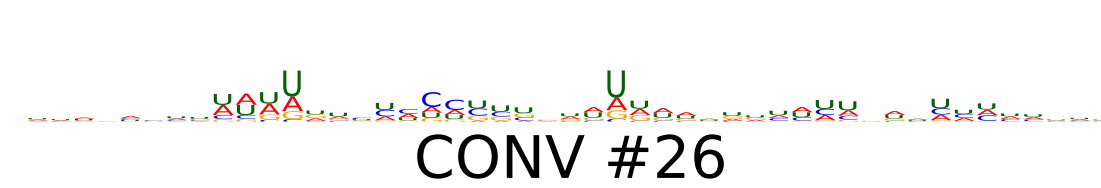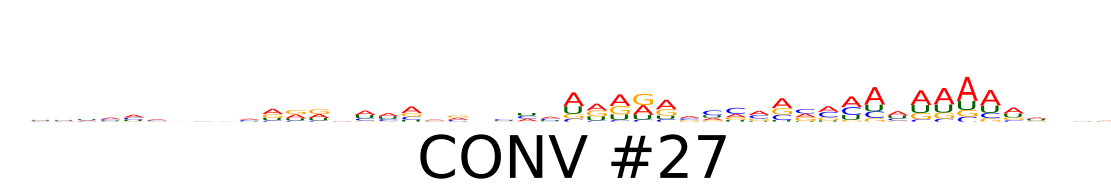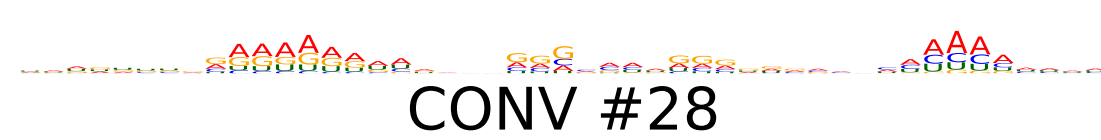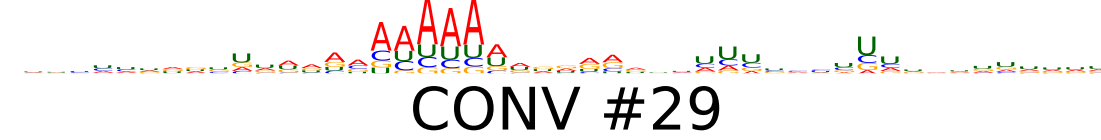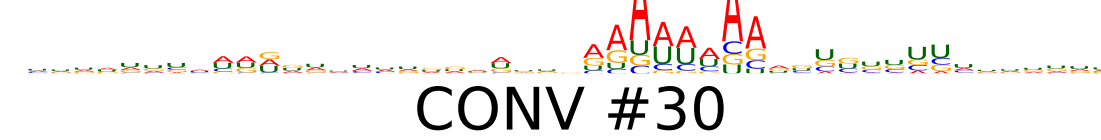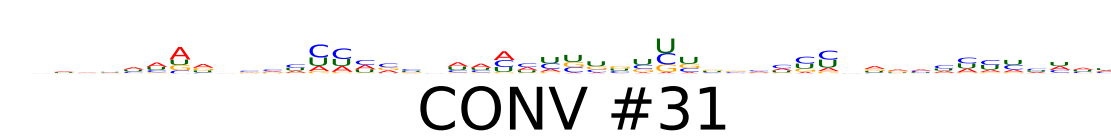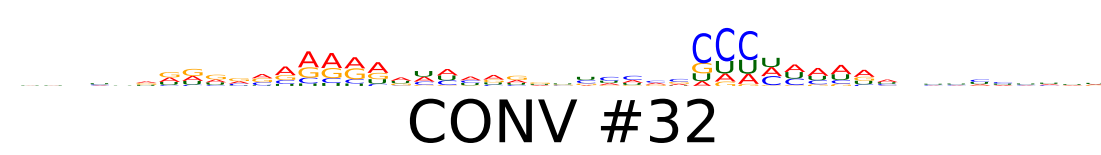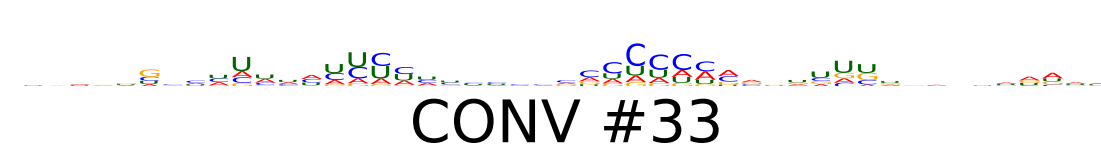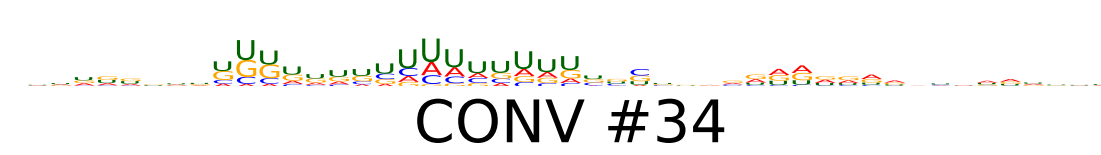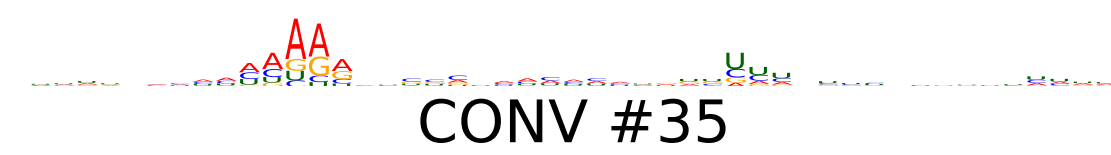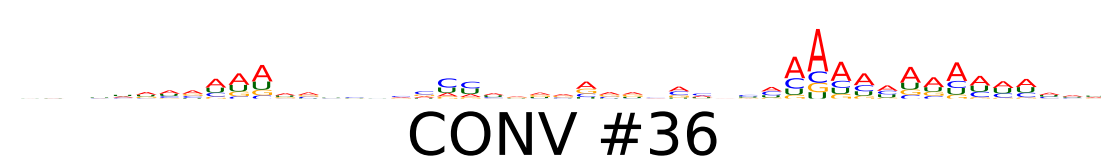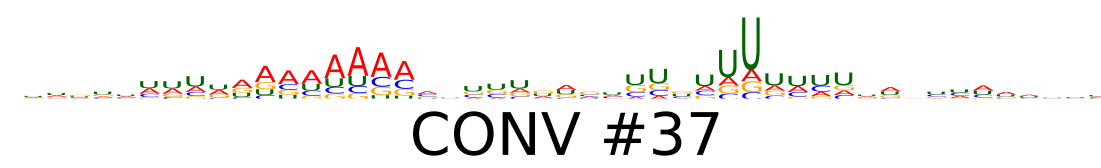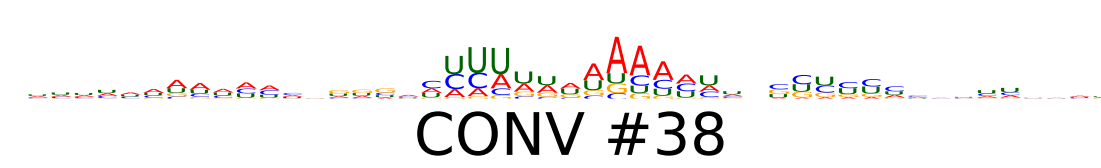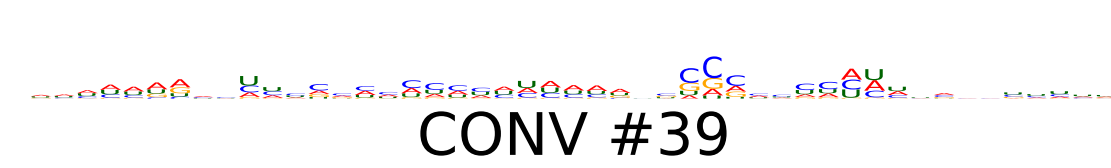

Supplement: Supplementary Figure S4 — Visualization of convolutional filters in layer 2 of DeeReCT-APA There are 40 convolutional filters in layer 2 of DeeReCT-APA. The model is trained on parental BL dataset and fine-tuned on F1. [file mmc5.pdf]
