# Supplementary material for: DeeReCT-APA: Prediction of Alternative Polyadenylation Site Usage Through Deep Learning
Source: Genomics Proteomics Bioinformatics. 2021 Mar 2;20(3):483–95. doi: 10.1016/j.gpb.2020.05.004 (PMC9801043; doi:10.1016/j.gpb.2020.05.004)
Supplement: Supplementary Table S1 — List of features used in Feature-Net and their corresponding dimensions [file mmc6.docx]

**Table S1 List of features used in Feature-Net and their corresponding dimensions**

| **Feature** | **Number of dims** |
| --- | --- |
| Polyadenylation Signals | 52 |
| AUE Elements | 12 |
| CUE Elements | 2 |
| ADE Elements | 12 |
| RBP Motifs | 72 |
| 1-mers | 16 |
| 2-mers | 64 |
| 3-mers | 256 |
| 4-mers | 992 |
| Nucleosome Occupancy | 12 |
| Position | 1 |
