# Supplementary material for: DeeReCT-APA: Prediction of Alternative Polyadenylation Site Usage Through Deep Learning
Source: Genomics Proteomics Bioinformatics. 2021 Mar 2;20(3):483–95. doi: 10.1016/j.gpb.2020.05.004 (PMC9801043; doi:10.1016/j.gpb.2020.05.004)
Supplement: Supplementary Table S2 — List of hyperparameters for the three DeeReCT-APA models [file mmc7.docx]

**Table S2 List of hyperparameters for the three DeeReCT-APA models**

| **Hyperparameter** | **DeeReCT-APA**  **(Single-Conv-Net)** | **DeeReCT-APA**  **(Multi-Conv-Net)** | **DeeReCT-APA**  **(Feature-Net)** |
| --- | --- | --- | --- |
| Batch size {16,32,64} | 32 | 32 | 32 |
| Learning rate [1e-5,1e-2] | 1e-3 | 1e-2 | 2e-5 |
| L2 weight decay [1e-5,1e-2] | 2e-5 | 1e-3 | 1e-5 |
| Conv filter width (layer 1) | 12 | 12 | - |
| Number of conv filters (Layer 1) | 16 | 40 | - |
| Pool width (Layer 1) | 12 | 3 | - |
| Conv filter width (Layer 2) | - | 12 | - |
| Number of conv filters (Layer 2) | - | 40 | - |
| Pool width (Layer 2) | - | 4 | - |
| LSTM input dim | 200 | 200 | 200 |
| LSTM output dim | 100 | 100 | 100 |
| Dropout rate [0.1,0.9] | 0.2 | 0.7 | 0.2 |

*Note:* For the hyperparameters that are sampled randomly, the ranges (in brackets) or sets (in braces) that they are sampled from are shown. The hyperparameters that are not applicable for a specific model are denoted by “-''.
