# Supplementary material for: DeeReCT-APA: Prediction of Alternative Polyadenylation Site Usage Through Deep Learning
Source: Genomics Proteomics Bioinformatics. 2021 Mar 2;20(3):483–95. doi: 10.1016/j.gpb.2020.05.004 (PMC9801043; doi:10.1016/j.gpb.2020.05.004)
Supplement: Supplementary Table S3 — Performance summary for the BL parental model and the F1 model fine-tuned from the BL parental model [file mmc8.docx]

**Table S3 Performance summary for the BL parental model and the F1 model fine-tuned from the BL parental model**

| Model |  | | | |
| --- | --- | --- | --- | --- |
|  | **MAE** | **Comparison Accuracy** | **Highest Usage Prediction Accuracy** | **Averaged Spearman’s Correlation** |
|  | **Performance on Parental Dataset** | | | |
| DeeReCT-APA (Feature-Net) | 17.60% ± 0.3% | 76.90% ± 0.9% | 61.50% ± 1.3% | 0.4999 ± 0.014 |
| DeeReCT-APA (Single-Conv-Net) | 17.42% ± 0.3% | 77.20% ± 0.7% | 62.22% ± 0.5% | 0.5035 ± 0.011 |
| DeeReCT-APA (Multi-Conv-Net) | **17.22% ± 0.3%** | **77.64% ± 0.4%** | **63.48% ± 0.9%** | **0.5140 ± 0.021** |
|  | **Performance on F1 Dataset** | | | |
| DeeReCT-APA (Multi-Conv-Net) | 18.20% ± 0.6% | 76.30% ± 1.6% | 61.10% ± 1.9% | 0.4661 ± 0.025 |
| Polyadenylation Code | 18.60% ± 0.6% | 74.94% ± 2.4% | 61.68% ± 1.8% | 0.4602 ± 0.027 |
| DeepPASTA | **17.80% ± 0.4%** | **77.14% ± 1.2%** | **64.52% ± 0.7%** | **0.4957 ± 0.009** |

*Note:* The table shows the performance of DeeReCT-APA with three different Base-Nets across four evaluation metrics. Results are shown in $\text{mean}\pm\text{std}$format. **A.** Performance on the Parental Dataset (BL) **B.** Performance on the F1 Dataset (fine-tuned from parental BL model). For Parental dataset, the values of MAE, Comparison accuracy, and Highest usage prediction accuracy for a random predictor are $43.12\%$, $50.00\%$ and $25.49\%$, respectively. For F1 dataset, they are $40.96\%$, $50.00\%$ and $28.56\%$, respectively.
