# Supplementary material for: DeeReCT-APA: Prediction of Alternative Polyadenylation Site Usage Through Deep Learning
Source: Genomics Proteomics Bioinformatics. 2021 Mar 2;20(3):483–95. doi: 10.1016/j.gpb.2020.05.004 (PMC9801043; doi:10.1016/j.gpb.2020.05.004)
Supplement: Supplementary Table S4 — Performance summary for the SP parental model and the F1 model fine-tuned from the SP parental model [file mmc9.docx]

**Table S4 Performance summary for the SP parental model and the F1 model fine-tuned from the SP parental model**

| Model |  | | | |
| --- | --- | --- | --- | --- |
|  | **MAE** | **Comparison Accuracy** | **Highest Usage Prediction Accuracy** | **Averaged Spearman’s Correlation** |
|  | **Performance on Parental Dataset** | | | |
| DeeReCT-APA (Multi-Conv-Net) | **17.90% ± 0.2%** | **76.20% ± 1.0%** | **61.58% ± 1.4%** | **0.4930 ± 0.018** |
| Polyadenylation Code | N/A | 74.98% ± 2.1% | 59.60% ± 2.4% | 0.4610 ± 0.026 |
| DeepPASTA | N/A | 72.12% ± 0.9% | 56.98% ± 1.3% | 0.4273 ± 0.011 |
|  | **Performance on F1 Dataset** | | | |
| DeeReCT-APA (Multi-Conv-Net) | **17.72%** ± **0.4%** | **76.52%** ± **1.5%** | **63.96%** ± **1.1%** | **0.4904** ± **0.007** |
| Polyadenylation Code | N/A | 74.62% ± 2.5% | 58.62% ± 1.8% | 0.4246 ± 0.030 |
| DeepPASTA | N/A | 69.22% ± 0.79% | 53.18% ± 2.7% | 0.3537 ± 0.029 |

*Note:* The table shows the performance of the three models across four evaluation metrics. Results are shown in $\text{mean}\pm\text{std}$ format. **A.** Performance on Parental Dataset (SP) **B.** Performance on F1 Dataset (fine-tuned from parental SP model). For Parental dataset, the values of MAE, Comparison accuracy, and Highest usage prediction accuracy for a random predictor are $43.12\%$, $50.00\%$ and $25.49\%$, respectively. For F1 dataset, they are $40.96\%$, $50.00\%$ and $28.56\%$, respectively.
