# Supplementary material for: DeeReCT-APA: Prediction of Alternative Polyadenylation Site Usage Through Deep Learning
Source: Genomics Proteomics Bioinformatics. 2021 Mar 2;20(3):483–95. doi: 10.1016/j.gpb.2020.05.004 (PMC9801043; doi:10.1016/j.gpb.2020.05.004)
Supplement: Supplementary Table S5 — Replicated experiments of 5-fold cross validation on 5 random splits [file mmc10.docx]

**Table S5 Replicated experiments of 5-fold cross validation on 5 random splits**

| Model |  | |  | | | |  | |
| --- | --- | --- | --- | --- | --- | --- | --- | --- |
|  | **Replicate 1** | **Replicate 2** | | **Replicate 3** | **Replicate 4** | **Replicate 5** | | **p-value** |
|  | **Comparison Accuracy on Parental Dataset** | | | | | | | |
| DeeReCT-APA (Multi-Conv-Net) | 77.64% | 77.53% | | 77.49% | 77.92% | 77.34% | | - |
| Polyadenylation Code | 75.88% | 75.39% | | 76.01% | 75.77% | 75.69% | | 1.6 × 10^−4^ |
| DeepPASTA | 74.08% | 73.94% | | 74.12% | 74.20% | 73.87% | | 4.5 × 10^−7^ |
|  | **Comparison Accuracy on F1 Dataset** | | | | | | | |
| DeeReCT-APA (Multi-Conv-Net) | 77.14% | 77.94% | | 76.88% | 77.09% | 77.10% | | - |
| Polyadenylation Code | 74.20% | 74.23% | | 74.15% | 74.02% | 74.31% | | 6*.*5 × 10^−5^ |
| DeepPASTA | 70.14% | 70.20% | | 70.87% | 71.02% | 70.46% | | 3*.*1 × 10^−5^ |

*Note:* The table shows the averaged comparison accuracy across the 5-fold cross validation of the three models on parental BL dataset and F1 dataset. There are 5 replicates for the experiment. At the end of each row, the table shows the p-value of the t-test of DeeReCT-APA's performance compared against the model of that row. **A.** Replicated Experiments on Parental Dataset (BL) **B.** Replicated Experiments on F1 Dataset
