# Supplementary material for: DeeReCT-APA: Prediction of Alternative Polyadenylation Site Usage Through Deep Learning
Source: Genomics Proteomics Bioinformatics. 2021 Mar 2;20(3):483–95. doi: 10.1016/j.gpb.2020.05.004 (PMC9801043; doi:10.1016/j.gpb.2020.05.004)
Supplement: Supplementary Table S6 — Comparison accuracy on dataset from Leung et al. 2018 [20] [file mmc11.docx]

**Table S6 Comparison accuracy on dataset from Leung et al. 2018 [20]**

| **Tissue** | **Comparison Accuracy** | | |
| --- | --- | --- | --- |
|  | **DeepPASTA** | **Polyadenylation Code** | **DeeReCT-APA**  **(Multi-Conv-Net)** |
| Brain | **0.908** | 0.895 | 0.895 |
| Breast | **0.900** | 0.886 | **0.900** |
| ES Cell | 0.910 | 0.911 | **0.925** |
| Ovary | 0.903 | 0.895 | **0.912** |
| SK Muscle | 0.906 | 0.893 | **0.914** |
| Testis | 0.893 | 0.856 | **0.905** |
| BCells1 | **0.905** | 0.896 | 0.904 |
| BCells2 | 0.901 | 0.893 | **0.907** |

*Note*: The performance of Polyadenylation Code and DeepPASTA is obtained from [20]and [21].
