# Supplementary material for: DeeReCT-APA: Prediction of Alternative Polyadenylation Site Usage Through Deep Learning
Source: Genomics Proteomics Bioinformatics. 2021 Mar 2;20(3):483–95. doi: 10.1016/j.gpb.2020.05.004 (PMC9801043; doi:10.1016/j.gpb.2020.05.004)
Supplement: Supplementary Table S7 — Replicated experiments of ablation study [file mmc12.docx]

**Table S7 Replicated Experiments of ablation study**

| Model |  | |  | | | |  | |
| --- | --- | --- | --- | --- | --- | --- | --- | --- |
|  | **Replicate 1** | **Replicate 2** | | **Replicate 3** | **Replicate 4** | **Replicate 5** | | **p-value** |
|  | **Comparison Accuracy on Parental Dataset** | | | | | | | |
| DeeReCT-APA  (Multi-Conv-Net)  (No Interaction Layer) | 76.12% | 76.04% | | 76.27% | 76.13% | 76.29% | | - |
| DeeReCT-APA  (Multi-Conv-Net)  (w/o BiLSTM) | 77.12% | 77.00% | | 77.21% | 77.29% | 76.94% | | 2.5 × 10^−6^ |
| DeeReCT-APA  (Multi-Conv-Net)  (BiLSTM) | 77.64% | 77.53% | | 77.49% | 77.92% | 77.34% | | 3.7 × 10^−3^ |
|  | **Comparison Accuracy on F1 Dataset** | | | | | | | |
| DeeReCT-APA  (Multi-Conv-Net)  (No Interaction Layer) | 76.28% | 76.33% | | 76.35% | 76.21% | 76.15% | | - |
| DeeReCT-APA  (Multi-Conv-Net)  (w/o BiLSTM) | 76.77% | 76.88% | | 76.87% | 77.16% | 76.49% | | 1*.*1 × 10^−3^ |
| DeeReCT-APA  (Multi-Conv-Net)  (BiLSTM) | 77.14% | 77.94% | | 76.88% | 77.09% | 77.10% | | 9*.*9 × 10^−2^ |

*Note:* The table shows averaged comparison accuracy across 5-fold cross validation of the two ablated models and the full DeeReCT-APA model on parental BL dataset and F1 dataset. There are 5 replicates for the experiment. At the end of each row, the table shows p-value of t-test of *the current row's* performance compared against the model of *the previous row*. In terms of most replicates, there is improvement over the previous row. **A.** Replicated Experiments on Parental Dataset (BL) **B.** Replicated Experiments on F1 Dataset
